# IFN-**λ** fine tunes protective immunity during mucosal viral infection

**DOI:** 10.1172/JCI211631

**Published:** 2026-10-15

**Authors:** Pablo Penaloza-MacMaster

Unlike the broadly expressed type I–IFN receptor, which sensitizes most cells in in the body to IFN-I, expression of the IFN-λ receptor is restricted to epithelial surfaces and select immune cell populations, allowing IFN-λ to limit viral replication mostly at epithelial barriers (1–5). IFN-λ can also provide antiviral protection, typically with less inflammation than type I IFN (6). Coordinated antiviral responses are particularly critical at mucosal or epithelial barriers, as these are the primary entry sites for many viruses, including herpes simplex virus type 1 and 2 (HSV-1 and -2), Epstein-Barr virus, human papilloma virus (HPV), and HIV-1, which can also induce detrimental inflammation. While the antiviral effects of IFN-λ at mucosal sites are well documented, the mechanism by which IFN-λ balances immune protection and immunopathology at mucosal sites is still not well understood.

In this issue of the *JCI*, Feng and colleagues investigated the antiviral and immunoregulatory functions of IFN-λ using genital HSV-2 infection in *Ifnar1*-deficient mice, which exhibit uncontrolled viral infection and severe immunopathology (7). Mucosal administration of IFN-λ shortly after viral infection reduced viral loads while also ameliorating immune-driven epithelial injury. Mechanistically, IFN-λ was shown to inhibit IL-6 production while rescuing production of CD4^+^ T cell–dependent IFN-γ. IFN-λ also enhanced the generation of monocyte-derived antigen presenting cells, resulting in improved T cell responses. Importantly, depletion of CCR2^+^ cells abrogated this effect, suggesting a critical role for CCR2^+^ cells. The findings suggest that IFN-λ can be an effective therapeutic candidate for improving antiviral responses at mucosal barrier sites, while tightly regulating detrimental inflammatory responses that often accompany viral infections.

Feng et al.’s study is important because robust antiviral protection and immunopathology (typically caused by T cell–mediated tissue damage) often occur in tandem, and their data suggest that IFN-λ can partially decouple these two seemingly inextricable processes, in part by regulating IL-6 and CD4^+^ T cell–dependent production of IFN-γ. In summary, IFN-λ may be best understood not simply as a less inflammatory IFN, but as an important immune regulator of mucosal antiviral immunity with therapeutic potential against mucosal infections.

## Conflict of interest

The author has declared that no conflict of interest exists.

## Version 1 10/15/2026

Electronic publication
